# Si-Miao-Yong-An Decoction Maintains the Cardiac Function and Protects Cardiomyocytes from Myocardial Ischemia and Reperfusion Injury

**DOI:** 10.1155/2021/8968464

**Published:** 2021-07-26

**Authors:** Wenwen Cui, Shen Xin, Lingjuan Zhu, Mingye Wang, Yuanyuan Hao, Yuqian Zhao, Yang Li, Yunlong Hou

**Affiliations:** ^1^College of Integrated Chinese and Western Medicine, Hebei University of Chinese Medicine, Shijiazhuang 050035, China; ^2^Department of Pharmacy, National Cancer Centre/Cancer Hospital, Chinese Academy of Medical Sciences and Peking Union Medical College, Beijing 100021, China; ^3^School of Traditional Chinese Materia Medica, Key Laboratory of Structure-Based Drug Design & Discovery of Ministry of Education, Shenyang Pharmaceutical University, Shenyang 110016, China; ^4^School of Life Science, Tsinghua University, Beijing 100084, China; ^5^Department of Cardiology, The Fourth Affiliated Hospital of Harbin Medical University/The Heilongjiang Academy of Medical Science, Harbin 150001, China

## Abstract

**Objective:**

The aim of this study was to determine whether Si-Miao-Yong-An decoction (SMYAD) could protect cardiomyocytes from ischemia/reperfusion (I/R) injury and its underlying mechanisms.

**Methods:**

C57BL/6 mice were used to establish a model of myocardial infarction by I/R injury and treated by SMYAD for 4 weeks. Then, the cardiac functions of mice were evaluated by cardiac magnetic resonance (CMR). Histopathological analysis for the heart remodeling was detected by H&E and Masson staining. The protein expression of collagen I, MMP9, and TNF*α* was detected by western blot in the heart tissues. H9C2 cells were used to establish the hypoxia/reoxygenation (H/R) model and SMYAD intervention. MTT assays detected the cell viability of myocardial cells. The expression level of IL-1*β* was evaluated by ELISA. The expression levels of LC3B-II/LC3B-I, p-mTOR, mTOR, NLRP3, procaspase 1, and cleaved-caspase 1 in H9C2 cells were evaluated by Western blot.

**Results:**

SMYAD improved cardiac functions such as ventricular volume and ejection fraction of the rats with ischemia/reperfusion injury. Morphological assay indicated that SMYAD reduced the scar size and inhibited fibrosis formation. It was found that SMYAD could regulate collagen I, MMP9, and TNF*α* protein expression levels in the heart tissues. SMYAD improved the survival rate of H9C2 cardiomyocytes in the H/R injury model. SMYAD elevated the rate of LC3B-II/LC3B-I protein expression, decreased the rate of p-mTOR/mTOR protein expression, and reduced expressions of caspase 1, NLRP3, and IL-1*β* in H/R cardiomyocytes.

**Conclusion:**

SMYAD exerted protective effects on ischemia/reperfusion injury in myocardial cells by activating autophagy and inhibiting pyroptosis. This might be the reason why SMYAD protected myocardial tissue and improved cardiac function in mice with ischemia/reperfusion.

## 1. Introduction

Myocardial infarction (MI) is an event of myocardial ischemia caused by coronary artery occlusion and interruption of blood flow leading to myocardial cell death [1], which retains a significant impact on global health and economic burden [2, 3]. Although timely reperfusion has proven to be the most effective treatment, the following ischemia/reperfusion (IR) injury indeed undermines the effectiveness and presented potential risks [4]. All efforts to seek ways and means of protecting cardiomyocytes from IR injuries are necessarily exerted with a pressing sense of urgency.

Programmed cell death (PCD) is a gene-directed way of cell self-death for normal cell turnover and tissue homeostasis, including apoptosis, autophagy, necroptosis, and pyroptosis, which occurs during the normal development of individuals and in abnormal physiological states or diseases [5–8]. The consensus among the pathological mechanisms of I/R injury is the programmed cell death of cardiomyocytes caused by histanoxia [2]. When myocardial I/R injury occurs, the increase of oxygen free radicals and calcium overload lead to massive death of myocardial cells and release endogenous danger molecules, as well as damage-associated molecular patterns (DAMPs), which will trigger and recruit abundance of inflammatory cells to clear cell debris away; in turn, undue inflammation aggravates the heart injury [9–11]. Along with researches going deep, DAMPs released from host cells can initiate and assemble cytosolic multiprotein oligomers called inflammasomes in immune cells such as macrophages. Initiation of the inflammasomes promotes cleavage and secretion of proinflammatory cytokines IL-1*β* and IL-18, as well as cleavage of Gasdermin D, which induces a distinct form of programmed cell death, referred to as pyroptosis [12–14]. Experimental evidence also suggests that pyroptosis is a primary mechanism to aggregate the cardiomyocyte death in the myocardial I/R injury [15]. Thus, the inhibition of pyroptosis holds promise as a therapeutic target for myocardial ischemia/reperfusion injury [16, 17]. In addition to the assembly of inflammasomes in the process of pyroptosis, the appearance of autophagosomes is also associated with PCD, referred to as autophagy. Recent researches have revealed that the activation of autophagy accompanied with PCD is an endogenic survival mechanism [18, 19]. In a sense, autophagy might counterbalance and regulate PCD to maintain the cell viability. There is a reasonable prospect that a therapeutic approach activating autophagy and inhibiting pyroptosis will be promising in protecting myocardiocytes from I/R injury.

Si-Miao-Yong-An decoction (SMYAD) is a traditional Chinese medicine formulation, which consists of *Lonicerae Japonicae Flos* (Jinyinhua), *Scrophulariae Radix* (Xuanshen), *Angelica Sinensis Radix* (Danggui), and *Glycyrrhizae Radix et Rhizoma* (Gancao) in a 3 : 3 : 2 : 1 proportion by weight. SMYAD first appeared in the “Hua Tuo Shen Yi Mi Zhuan” of the Eastern Han Dynasty and was edited in the “Yan Fang Xin Bian” of the Qing Dynasty. SMYAD was also listed as one of the 100 classic prescriptions in the “Catalogue of Ancient Classic Prescriptions (the First Batch),” which was issued by the National Administration of Traditional Chinese Medicine in 2018. This famous ancient recipe was traditionally used for gangrene and in modern medicine to treat peripheral vascular diseases, diabetes, coronary heart disease, and heart failure [20]. According to reports, SMYAD has been reported to exert pharmacological effects, including anti-inflammatory, regulating angiogenesis, antioxidative stress, regulating blood lipids, and improving blood rheology [21–23]. Previous study identified 16 compounds in the SMYAD in rat plasma using UPLC-Q/TOF-MS, most of which showed cardiomyocyte protective activity [20]. However, the effect of SMYAD in myocardial I/R injury has yet not been explored.

In this study, we performed *in vivo* I/R injury mouse model to prove the protective effects of SMYAD on myocardial injuries and carried out studies on the mechanism of actions *in vitro*. The main objective of this study is to investigate the pharmacological effects of SMYAD on MI and provide the experimental basis for clinical application.

## 2. Materials and Methods

### 2.1. Materials

The materials were sodium chloride injection (Harbin Sanlian Pharmaceutical, China); Dulbecco's modified Eagle's medium (DMEM) (Gibco, USA); fetal bovine serum (Gibco, USA); penicillin/streptomycin (Gibco, USA); 0.25% (w/v) Trypsin-0.53 mM EDTA (Gibco, USA); glucose-free DMEM (Gibco, USA); IL-1 *β* ELISA Kit (Elabscience, China); BCA Assay Kit (TDY Biotechnology Co., Ltd., China); anti-GAPDH antibody (CST, #5174); anti-Collagen I antibody (Abcam, ab270993); anti-MMP9 antibody (Abcam, ab38898); anti-TNF*α* antibody (CST, #3707); anti-*β*-actin antibody (CST, #3700s); anti-LC3B antibody (Abcam, ab192890); anti-p-mTOR antibody (CST, #5536); anti-mTOR antibody (CST, #2983); anti-NLRP3 antibody (Proteintech, 19771-1-AP); anti-caspase 1 antibody (CST, #2225); goat anti-mouse IgG H&L (IRDye® 800CW) (ab216772, 1: 5000), and goat anti-rabbit IgG H&L (IRDye® 680RD) preadsorbed (ab216777, 1: 5000).

### 2.2. Animals

6–8-week-old male *Kunming* mice (18–22 g) were purchased from Beijing Huafukang Biotechnology Co., LTD (certificate no.: SYXK (Jing) 2019–0008, Beijing, China). Animals were maintained on a 14 h/10 h light/dark cycle with temperature of 20°C ± 2°C, and food and water were available ad libitum. The experimental procedures and animal welfare were in accordance with the Ethical Regulations on the Care and Use of Laboratory Animals of Harbin Medical University. Animals were acclimatized for three days and randomly divided into four groups (*n* = 12 per group): control group (Con), myocardial ischemia/reperfusion (MI/R) injury model group (I/R), myocardial ischemia/reperfusion (MI/R) injury + Si-Miao-Yong-An decoction 12 g/kg/day (SMYAD-L), and myocardial ischemia/reperfusion (MI/R) injury + Si-Miao-Yong-An decoction 24 g/kg/day (SMYAD-H).

### 2.3. Establishment of Mice Myocardial Ischemia/Reperfusion (MI/R) Injury Model and Drug Treatment

**Figure 1:**
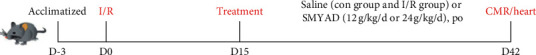
Timeline graph of animal experiments.

The myocardial infarction animal model was established by left anterior descending (LAD) coronary artery ligation. Mice were anaesthetized by 2% isoflurane inhalation, and the skin in the operating area of mice was prepared. Endotracheal intubation was used to assist breathing and electrocardiogram was recorded. The chest was opened by a vertical cut, lateral to the left side of the sternum, the heart was temporarily exteriorized through a left thoracic incision, and a 7-0 silk suture slipknot was placed around the left anterior descending coronary artery. The chest clamp was used to temporarily close the chest, and electrocardiograph (ECG) was recorded to observe whether the ST segment was elevated. After 45 min of ischemia, the slipknot was released, allowing the myocardial reperfusion. 5-0 polypropylene sutures were used to close the ribcage and the muscle layer in turn. After removal of the tracheal intubation tube, the animal was placed in a clean recovery cage under a heat lamp. Control group mice underwent the same operation except that the suture placed around the LAD was not tied. On the 15th day after modeling, all mice except those in the Con group and I/R group were orally treated with saline, 10 ml/kg/day, and the mice in the SMYAD-L group and SMYAD-L group were continuously administrated with Si-Miao-Yong-An decoction (at a dose of 12 g/kg/d or 24 g/kg/d, corresponding to the 4 g/kg/d and 8 g/kg/d extracts, respectively) for 28 days, as shown in Figure 1.

### 2.4. Cardiac Functions Detected by Cardiac Magnetic Resonance (CMR)

After 28 days of continuous administration, Cardiac Magnetic Resonance (CMR) detected cardiac function, including left ventricular end-diastolic volume (LV-EDA), left ventricular end-systolic volume (LV-ESV), LV-ligation zone thickness, downstream thickness of LV-ligation zone, and ejection fraction (EF). The CMR examinations were performed on a 9.4 T preclinical scanner (Bruker, BioSpec 94/20 USR TT), equipped with 660 mT/m gradients (slew rate: 4200 T/m/s) and three circular polarized mouse body volume coils with the inner diameters of 10 mm, 20 mm, and 30 mm. Mice were anaesthetized (isoflurane, 2.5% for induction and 1.5% for maintenance, 2 L/min oxygen). Respiration and body temperature were monitored during CMR (SA Instruments, Inc., Stony Brook, NY, USA) and maintained at about 30 breaths-per-minute and 37°C, respectively. CMR protocol included the following: precontrast long-axis 4-chamber and long-axis 2-chamber 2D intragate cine FLASH sequences (repetition time = 30 ms, echo time = 2.5 ms, number of repetitions = 1, field of view = 38.4 × 38.4 mm, slice thickness = 0.8 mm, and total scan time = 1 min 36 s) which were used to plan the short-axis ce-3D-IG-cine stack (repetition time = 7 ms, echo time = 2.423 ms, number of repetitions = 1, flip angle = 15°, slice thickness = 1 mm, field of view = 20 × 20 mm, and total scan time = 18 min 40 s) encompassing the entire LV from the base to the apex.

### 2.5. Histopathological Analysis

Mice were sacrificed and the hearts were harvested on the 30th day after the drug treatment. 10%-formaldehyde-fixed heart tissues were embedded in paraffin and later sectioned onto the glass slides (4 *μ*m). The heart tissue sections were stained with hematoxylin and eosin (H&E) to assess for myocardial I/R injury and with Masson's trichrome staining to assess for fibrosis in the heart.

### 2.6. Cells Culture for H9C2 Cell Line

H9C2 cell line was purchased from American Type Culture Collection (ATCC). H9C2 cells were maintained in complete medium (Dulbecco's modified Eagle's medium (DMEM) supplemented with 10% fetal bovine serum (FBS) and 1% penicillin/streptomycin) and incubated in a humidified 5% CO_2_ incubator at 37°C. The medium was replaced every 2–3 days, and the cells were digested with 0.25% (w/v) Trypsin-0.53 mM EDTA when the density of the cells reached approximately 80–90% confluent. A subcultivation ratio of 1 : 2 to 1 : 4 is recommended.

### 2.7. Establishment of Hypoxia/Reoxygenation (H/R) Model *In Vitro* and Drug Treatment

When the H9C2 cells grew to approximately 70–80% confluence, they were synchronized by fresh DMEM media without serum or antibiotics, and then the medium was removed from the synchronized H9C2 cells. H9C2 cells were inoculated into 96-well plates or 6-well plates and treated for the subsequent experiments. When the density of cells reached approximately 50–60% in the plates, the hypoxia/reoxygenation (H/R) model group cells were incubated for 4 h with serum and glucose-free medium in the three-gas incubator (NuAire, NU-5741E, USA) with a mixture of 94% N_2_, 5% CO_2_, and 1% O_2_ at 37°C. Subsequently, the cells were removed to the complete medium and normoxic chamber for 20 h to establish reoxygenation.

The experimental groups were divided into Si-Miao-Yong-An decoction (SMYAD) group and Si-Miao-Yong-An decoction under hypoxia/reoxygenation (H/R + SMYAD) group. At the beginning of reoxygenation, the drug was added to the completed medium according to the SMYAD concentration gradient (0, 50, 75, 100, 125, and 150 *μ*g/mL).

### 2.8. Cell Viability Assays

Cell viability was determined by the Methyl Thiazolyl Tetrazolium (MTT) assays. Cells were seeded at 1 × 10^5^ cells/well in 96-well plates overnight. After the treatment of different culture environment or drug concentration, cells were incubated with 50 ul 5 mg/mL MTT for 2–4 h and subsequently solubilized in 150 *μ*L DMSO. Cell viability was assessed by measuring the absorbance at 490 nm by using a microtiter plate reader (Tecan, Switzerland).

### 2.9. Enzyme-Linked Immunosorbent Assay (ELISA) for Interleukin-1*β* (IL-1*β*)

Cells culture supernatants of 96-well plates were collected for assessing the concentrations of IL-1*β* in the supernatants by ELISA according to the manufacturer's instructions. Briefly, the cell culture media were centrifuged at 2,000 ×g for 10 minutes to remove debris. All reagents, samples, and standards were prepared following the instructions. The 50 *μ*L of sample and 50 *μ*L of Antibody Cocktail were added to the 96-well plate and incubated for 1 hour at room temperature on a plate shaker. Then, the 96-well plates were washed three times, 100 *μ*L of TMB development solution and 100 *μ*L of Stop Solution were added, and the OD at 450 nm was recorded by the microtiter plate reader (Tecan, Switzerland).

### 2.10. Western Blot

Total proteins in H9C2 cells or heart tissues were extracted by RIPA lysis buffer with 0.1% phenylmethanesulfonyl fluoride (PMSF) at indicated time points. BCA kit detected total protein concentration, and the protein concentration was homogenized. The protein samples were separated on 4–20% precast gels at 150 V for 1 h and transferred on nitrocellulose filter (NC) membranes at 110–120 V for 50 min. The membrane was blocked for 30 min at room temperature in blocking buffer to reduce nonspecific binding. After blocking, the membranes were incubated with primary antibodies (collagen I, 1 : 1000; MMP9, 1 : 1000; TNF*α*, 1 : 1000; LC3B, 1 : 1000; p-mTOR, 1 : 1000; mTOR, 1 : 1000; NLRP3, 1 : 500; procaspase 1, 1 : 1000; cleaved-caspase 1, 1 : 1000; GAPDH, 1 : 1000; and *β*-actin, 1 : 1000) at 4°C overnight. After washing with TBS containing 0.1% Tween 20, the membranes were followed by 1 h incubation with fluorescent secondary antibodies at 37°C.

### 2.11. Statistical Analysis

The results were presented as the means ± standard deviation. All analyses were performed using SPSS 19.0 statistical software. All statistics and data evaluation were subjected to statistical analysis using one-way ANOVA. ^#^*p* < 0.05, ^##^*p* < 0.01, ^*∗*^*p* < 0.05, ^∗∗^*p* < 0.01, and ^∗∗∗^*p* < 0.001 were considered significant.

## 3. Results

### 3.1. The Effect of SMYAD on Cardiac Function Index of I/R Mice Model

**Figure 2:**
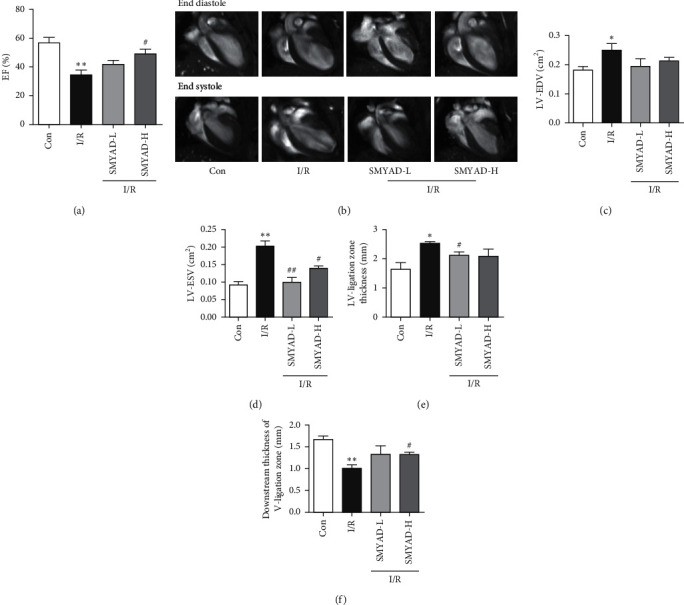
The coronal view diagram of CMR and the effect of SMYAD on cardiac functions of I/R mice model (*n* = 3). LA: left atrial; LV: left ventricular; RV: right ventricular. LV-EDA: left ventricular end-diastolic volume; LV-ESV: left ventricular end-systolic volume; EF: ejection fraction. ^*∗*^*p* < 0.05 and ^∗∗^*p* < 0.01 versus control group; ^#^*p* < 0.05 and ^##^*p* < 0.01 versus I/R group.

To evaluate the effect of SMYAD in I/R mice model, Cardiac Magnetic Resonance (CMR) was performed to observe the cardiac function index changes. As shown in Figure 2(b), in the coronal view diagram of CMR, it was found that the inner diameter of the left ventricle was significantly increased at the end of diastole and end of systole, and the ventricle was severely dilated in the I/R model group, while the left ventricular cavity was decreased in the SMYAD-L and SMYAD-H group after I/R modeling. Compared with the control group, LV-EDA, LV-ESV, and LV-ligation zone thickness were significantly increased, and EF and downstream thickness of LV-ligation zone were significantly decreased in the H/R group. Compared with the I/R group, both SMYAD-L and SMYAD-H improved myocardial infarction symptoms and cardiac function to varying degrees (Figures 2(a) and 2(c)–2(f)).

The Effect of SMYAD on Pathological Changes of I/R Mice Model in the Heart Tissue

**Figure 3:**
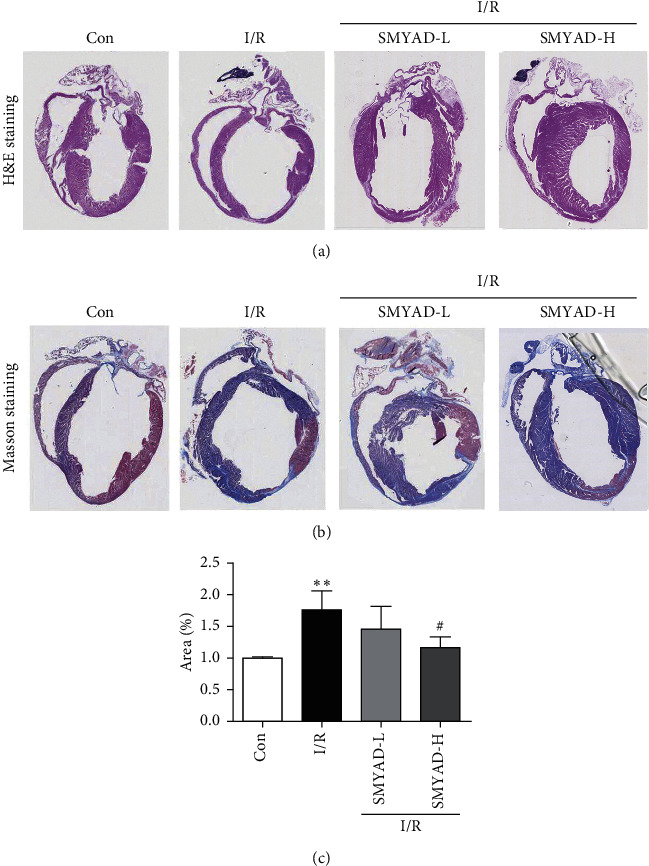
The effect of SMYAD on pathological changes of I/R mice model in the heart tissue (*n* = 3). (a, b) Representative images of H&E staining and Masson staining in the whole heart, respectively. Scale bar is 1 mm. (c) The results of the quantitative analysis of Masson staining using ImageJ.

The study evaluated the changes in cardiac structure and myocardial tissue by histopathological staining: hematoxylin and eosin (H&E) and Masson's trichrome staining. According to H&E staining, the myocardium of the control group was dyed evenly with normal myofibrils with a neat arrangement and myocardial cells were in order. In the I/R model group, the cardiac cavity of mice was significantly enlarged and the myocardial infarction area was visible. In addition, necrosis of massive myocardial cells, muscle fibers dissolution, and deposition of the cell matrix were observed. These changes of cardiac structure markedly improved in the SMYAD groups, especially the SMYAD-H group (Figure 3(a)). According to Masson staining, the myocardial infarction and severe fibrosis were observed with increased blue scar tissue at the edge of the infarction area in the I/R model group. The myocardial infarction symptoms and fibrosis response in both SMYAD groups were alleviated (Figure 3(b)).

### 3.2. The Effect of SMYAD on Expression of Collagen I, MMP9, and TNF*α* Protein in the Heart Tissue of I/R Mice Model

**Figure 4:**
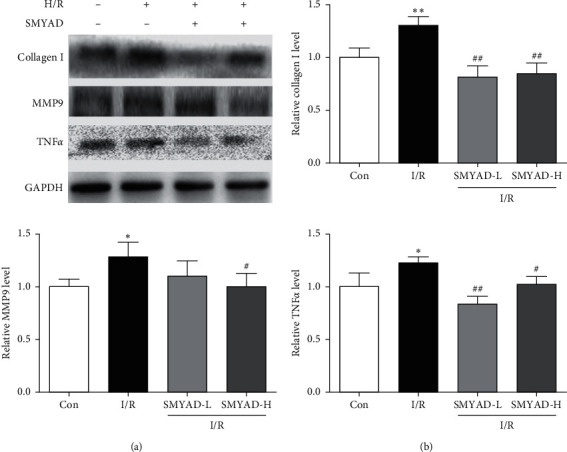
The effect of SMYAD on the expressions of collagen I, MMP9, and TNF*α* protein in the heart tissue of I/R mice model (*n* = 3). (a, b) Protein levels of collagen I, MMP9, and TNF*α* in each group by western blot. ^*∗*^*p* < 0.05 and ^∗∗^*p* < 0.01 versus control group; ^#^*p* < 0.05 and ^##^*p* < 0.01 versus I/R group.

The Western blot is used for the expressions of Collagen I, MMP9, and TNF*α* protein in the heart tissue of I/R mice model. As shown in Figure 4, the relative expression of each lane was normalized by the control group in the first lane. The results showed that the expressions of Collagen I, MMP9, and TNF*α* protein in the model group were higher than those in the control group, and the SMYAD-H and SMYAD-L groups decreased the expressions of Collagen I, MMP9, and TNF*α* protein after I/R.

### 3.3. The Protective Effect of SMYAD on Myocardial Cells Injured by H/R Model

**Figure 5:**
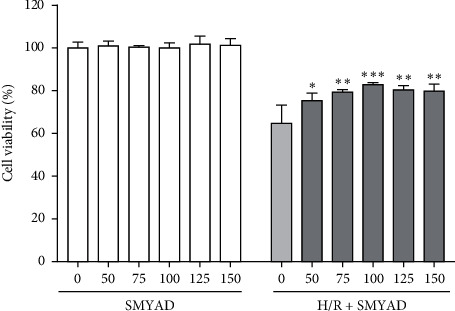
The protective effect of different doses of SMYAD (0, 50, 75, 100, 125, and 150 *μ*g/mL) on the viability of cultured H9C2 cardiomyocytes under the normal and H/R conditions (*n* = 6), as determined by the MTT assay kit. ^*∗*^*p* < 0.05, ^∗∗^*p* < 0.01, and ^∗∗∗^*p* < 0.001 versus H/R group.

Under normal culture conditions, SMYAD did not affect the viability of H9C2 cells in the concentration range of 50–150 *μ*g/mL. Under hypoxia/reoxygenation model conditions, SMYAD increased cell viability in a concentration-dependent manner in the range of 50–100 *μ*g/mL (Figure 5). The results showed that SMYAD had a protective effect on cardiomyocytes injured by hypoxia/reoxygenation in a dose-dependent manner from 50 to 150 *μ*g/ml.

### 3.4. The Effect of SMYAD on Autophagy of Myocardial Cells Injured by H/R Model

**Figure 6:**
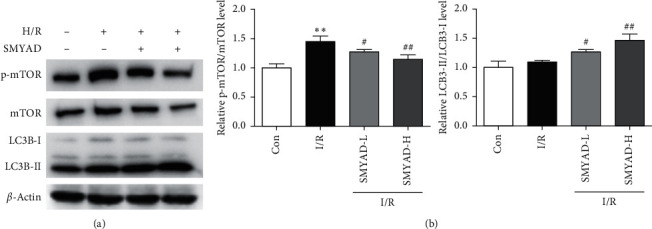
The effect of SMYAD on autophagy of myocardial cells injured by H/R model. (a, b) Protein levels of p-mTOR, mTOR, and LC3B-I/II in each group by western blot (*n* = 3). ^∗∗^*p* < 0.01 versus control group; ^#^*p* < 0.05 and ^##^*p* < 0.01 versus I/R group.

Western blot analysis demonstrated the LC3B-I, LC3B-II, mTOR, and p-mTOR protein levels from the total protein of cardiomyocytes, and the relative expression of each sample was normalized by the control group lane. Compared with the control group, the rate of p-mTOR/mTOR was significantly increased in the H/R group. Compared with the H/R group, the rate of p-mTOR/mTOR was significantly decreased, and LC3B-II/LC3B-I was significantly increased in the SMYAD-L (75 *μ*g/mL) and SMYAD-H (150 *μ*g/mL) groups (Figure 6).

### 3.5. The Effect of SMYAD on Pyroptosis of Myocardial Cells Injured by H/R Model

**Figure 7:**
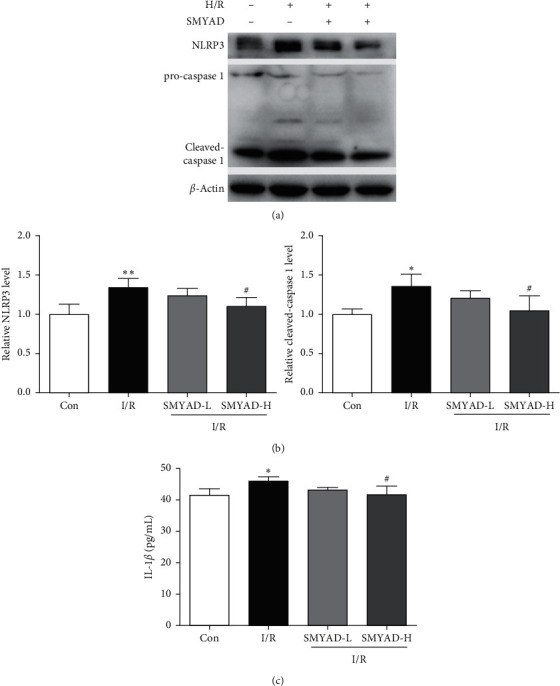
The effect of SMYAD on pyroptosis of myocardial cells injured by H/R model. (a, b) Protein levels of NLRP3, procaspase 1, and cleaved-caspase 1 in each group by western blot (*n* = 3). (c) Expression level of IL-1*β* by ELISA in each group. ^∗∗^*p* < 0.01 versus control group; ^#^*p* < 0.05 versus I/R group.

Western blot analysis demonstrated the NLRP3, procaspase 1, and cleaved-caspase 1 protein levels and ELISA analysis detected the expression level of IL-1*β*. Compared with the control group, the expressions of NLRP3, procaspase 1, and cleaved-caspase 1 were significantly increased in the H/R group. Compared with the H/R group, the expressions of NLRP3, procaspase 1, and cleaved-caspase 1 were decreased in the SMYAD-L (75 *μ*g/mL) and SMYAD-H (150 *μ*g/mL) groups (Figure 7).

## 4. Discussion

MI is characterized by massive myocardial cell death and has a highly likely poor prognosis [24]. Although prevention and treatment works have been greatly improved, myocardial infarction still has a significant impact on global health and is also the main cause of mortality worldwide [25]. The therapeutic regimes for MI contain thrombolytic therapy, coronary intervention therapy, and drug therapy. All approaches are aimed at restoring blood supply to the ischemic zone and replenishing nutrients and oxygen [26, 27]. However, concerns have been raised about the lack of treatments on the subsequent I/R injury [28, 29].

Accumulating evidence has proved that SMYAD can improve heart function and alleviate cardiac fibrosis in the mice models of heart failure [22, 23, 30]. In this study, a mouse MI model induced by I/R injury was performed to evaluate the cardioprotective effects of SMYAD. After 4 weeks of treatment, the functional test for the hearts of mice was performed by CMR. We found that there was not much difference in EDV among model and SMYAD groups, but the difference in ESV among them was significant. Moreover, the left ventricular wall downstream to the ligation position was thicker in the mice of SMYAD groups than that in the model group, and the EF of left ventricle in the mice of SMYAD group was also higher than that in the model group (Figure 2). All these results suggested that 4 weeks of application of SMYAD effectively maintained the cardiac functions of I/R injury by the inhibition of cardiac remodeling and the reduction of myocardiocytes loss. The following H&E and Masson staining indicated that 4 weeks of application of SMYAD reduced the infarct sizes and inhibited the myocardial fibrosis (Figure 3). When MI occurs, inflammation mediates myocardial fibrosis [31] and the abnormal proliferation of collagen in the myocardial interstitium [32], which seriously affects cardiac function. Matrix metalloproteinases (MMPs) act as targets of myocardial fibrosis to affect the process of myocardial fibrosis [33]. SMYAD reduced the expression of heart failure markers and fibrosis markers (collagen I, MMP9, and TNF*α*) in the heart tissue of model animals after myocardial infarction (Figure 4). To prove the hypothesis that the therapeutic effects of SMYAD in MI were partially dependent on the protective capabilities of cardiomyocytes, the *in vitro* [34, 35] study of oxygen-glucose deprivation (OGD) in H9C2 cardiomyocytes was performed to mimic the H/R injury model. The results indicated that SMYAD protected myocardial cells from OGD in a dose-dependent manner (Figure 5).

The mechanisms of myocardial I/R injury are intricate, including calcium overload, oxygen free radicals, and inflammatory response, eventually triggering the process of PCD in hearts [36–39]. Pyroptosis, as one of PCD forms, is called inflammatory cell death and mainly depends on the formation of inflammasome NLRP3 that activates caspase 1 to release IL-1*β* and IL-18 into the extracellular environment, recruiting inflammatory cells to aggregate the inflammatory response [13, 14]. We found that SMYAD reduced the expressions levels of NLRP3 and caspase 1 in the OGD of H9C2 cardiomyocytes. The concentration of IL-1*β* in the culture supernatant among groups was detected by ELISA, and SMYAD reduced the IL-1*β* yield in a dose-dependent manner. Having demonstrated that SMYAD targeted and inhibited the pyroptosis pathway, we next sought to identify the counterbalancing mechanism. When myocardial I/R injury occurs, autophagy will be activated. On the one hand, autophagy helps to clear damaged organelles and proteins and on the other hand provides energy for ischemic cells [40, 41]. Studies have shown that maintaining autophagy flux during ischemia/reperfusion can reduce infarct size and protect cardiomyocytes [42, 43]. The mTOR signaling pathway is considered an important signaling pathway during cardiac I/R injury. Studies have shown that the decrease of phosphorylated mTOR level may lead to the activation of autophagy [44]. The change of microtubule-associated protein 1A/1B-light chain 3B (LC3B-II) is a well-established marker of autophagosome formation. In this study, we found that SMYAD elevated the protein expression of LC3B-II and the rate of p-mTOR/mTOR in the OGD of H9C2 cardiomyocytes, which suggested that the antipyroptosis of SMYAD resulted from the activation of endogenous autophagy.

## 5. Conclusions

In this study, SMYAD protected myocardial tissue and improved cardiac function in the mouse MI model induced by I/R injury. Moreover, SMYAD exerted effects on cardiomyocyte protection by activating autophagy and inhibiting pyroptosis *in vitro*. These findings support the clinical application of SMYAD for patients who suffer from MI, especially in preventing the myocardial tissue from I/R injury.

## Data Availability

The data used to support the findings of this study are available from the corresponding author upon request.

## Abbreviations

SMYAD: Si-Miao-Yong-An decoction
CMR: Cardiac magnetic resonance
H/R: Hypoxia/reoxygenation
MI: Myocardial infarction
IR: Ischemia/reperfusion
PCD: Programmed cell death
DAMPs: Damage-associated molecular patterns
DMEM: Dulbecco's modified Eagle's medium
LAD: Left anterior descending coronary artery
MI/R: Myocardial ischemia/reperfusion
ECG: Electrocardiograph
H&E: Hematoxylin and eosin
ATCC: American Type Culture Collection
FBS: Fetal bovine serum
MTT: Methyl thiazolyl tetrazolium
ELISA: Enzyme-linked immunosorbent assay
IL: Interleukin
PMSF: Phenylmethanesulfonyl fluoride
NC: Nitrocellulose filter
LV-EDA: Left ventricular end-diastolic volume
LV-ESV: Left ventricular end-systolic volume
EF: Ejection fraction
OGD: Oxygen-glucose deprivation.
